# Awareness of idiopathic intracranial hypertension in children among pediatricians in Saudi Arabia: a cross-sectional study

**DOI:** 10.3389/fped.2025.1675107

**Published:** 2025-11-07

**Authors:** Mohammed A. Al-Omari, Ahmad A. AlShammari, Hwazen A. Shash, Abdullah K. Almutairi, Fai A. AlQahtani, Razan H. AlAttas, Somyyah I. AlNaimi, Alia M. AlAmmari, Sulaiman Almobarak

**Affiliations:** 1 Department of Pediatrics, College of Medicine, Imam Abdulrahman Bin Faisal University, Dammam, Saudi Arabia; 2 Pediatric Neurology Department, King Saud Medical City, Riyadh, Saudi Arabia; 3 College of Medicine, Alfaisal University, Riyadh, Saudi Arabia

**Keywords:** idiopathic intracranial hypertension, pseudotumor cerebri, headache in children, awareness, diagnosis, pediatricians

## Abstract

**Background:**

Idiopathic intracranial hypertension (IIH) is a rare condition in children, often underdiagnosed due to its rarity and non-specific clinical presentations. This study aimed to assess the awareness and knowledge of pediatricians in Saudi Arabia regarding IIH in the pediatric population.

**Methods:**

A nationwide cross-sectional study was conducted using a validated electronic questionnaire distributed to registered pediatricians across various subspecialties. The questionnaire assessed knowledge on IIH definition, clinical presentation, diagnosis, management, and prognosis. Participants were categorized by years of experience into: Group A (1–9 years) and Group B (≥10 years). Responses between the two groups were compared.

**Results:**

A total of 234 pediatricians completed the questionnaire (Group A: 43%, *n* = 101; Group B: 57%, *n* = 133). The overall knowledge level was moderate, with a correct response rate of 73%. While most respondents demonstrated understanding of IIH definitions and risk factors, knowledge gaps were noted in incidence, diagnostic criteria, and management. Pediatricians in Group B were significantly more likely to recognize headache as the most common symptom (*p* = 0.003) and the importance of a multidisciplinary approach to management (*p* = 0.001).

**Conclusion:**

The study identified significant knowledge gaps among pediatricians regarding IIH in children, particularly among those with fewer years of clinical experience. Addressing these gaps through targeted educational programs is essential to enhance early recognition, accurate diagnosis, and effective management of pediatric IIH, ultimately reducing the risk of serious complications such as irreversible vision loss.

## 1 Introduction

Idiopathic intracranial hypertension (IIH), previously known as pseudotumor cerebri, is a rare neurological condition characterized by elevated intracranial pressure (ICP) with no evidence of an underlying pathology (1). Although IIH is more frequently reported in young adults, it can also occur in children and adolescents. The incidence of IIH in children has been estimated to range from 0.5 to 2.3 per 100,000 children (2, 3). Though regional data are limited, a study from Oman reported an incidence of 1.9 per 100,000 children under the age of 15, with a higher incidence among females (4). Moreover, obesity is a well-recognized risk factor for IIH, although the exact mechanisms underlying this association is not well-understood (5).

Saudi Arabia, like many other countries, has seen a rise in the number of reported pediatric IIH cases in recent years (6, 7). This trend is particularly concerning given the high and rising prevalence of childhood obesity, which is a known modifiable risk factor for IIH. A recent study reported that 20.6% of children aged 2–19 years in Saudi Arabia are overweight or obese (8). It is therefore plausible that the actual incidence of pediatric IIH in Saudi Arabia is likely higher than reported in regions with lower obesity rates.

The modified Dandy criteria is used to establish the diagnosis of IIH. The criteria include the presence of signs and symptoms of elevated ICP, elevated lumbar puncture opening pressure with normal cerebrospinal fluid (CSF) composition, normal neuroimaging findings, and a normal neurologic examination apart from sixth cranial nerve palsy (3). Subsequent revisions of the criteria have allowed for the inclusion of papilledema or specific neuroimaging findings in the absence of papilledema (9). Although well-established diagnostic criteria exist, IIH in children is often underrecognized or misdiagnosed due to its rarity and non-specific clinical features, potentially leading to delayed treatment and potential serious complications including loss of vision (7).

In clinical practice, pediatricians play a critical role in the early identification and referral of children with suspected IIH. However, there is limited data on pediatricians' awareness and understanding of IIH, particularly in Saudi Arabia. The present study aimed to assess pediatricians' knowledge of IIH in children within the Saudi healthcare context. By identifying gaps in awareness, this study seeks to inform future educational initiatives and improve the early recognition and management of pediatric IIH.

## 2 Materials and methods

### 2.1 Study design

This study is a cross-sectional, questionnaire-based observational study. The study was conducted between July and November 2023. The questionnaire was initially developed by the authors based on current clinical knowledge of idiopathic intracranial hypertension (IIH). It was then reviewed by three expert pediatric neurologists (not among the authors), and their feedback was used to refine the content for accuracy and clinical relevance. Following this expert review, a pilot study was conducted with 52 pediatric practitioners from diverse institutions and subspecialties to assess the clarity and validity of the instrument. The pilot responses were excluded from the final analysis.

The final version of the questionnaire was electronically distributed through the Saudi Commission for Health Specialties (SCFHS) portal. The target population comprised 10,275 registered pediatricians in the SCFHS database, of whom 2,500 were randomly selected to receive the invitation. Each participant could respond only once. All pediatric practitioners registered with SCFHS were eligible to participate. Pediatric neurologists were excluded to avoid potential bias.

The overall knowledge level was categorized as low (<50%), moderate (50%–75%), or high (>75%), consistent with prior awareness studies.

Electronic informed consent was obtained from all participants before proceeding with the survey.

A total of 234 pediatricians completed the questionnaire without missing responses, yielding a response rate of 9.36% (234/2,500). Participants were stratified into two groups based on their total years of professional practice in pediatrics following medical graduation: Group A (1–9 years) and Group B (10 years or more). Responses from both groups were compared to evaluate differences in awareness, diagnostic approaches, and management of pediatric IIH.

### 2.2 Questionnaire

The questionnaire consisted of two main sections. The first section gathered demographic data, including age, gender, academic rank, years of experience, and subspeciality. The second section included statement-based questions addressing various aspects of IIH: definition, incidence, risk factors, clinical presentation, diagnostic criteria, management, and prognosis. Responses were collected using a three-point agreement scale: “I agree”, “I don't agree”, or “I don't know”.

Cronbach's alpha was calculated for internal consistency based on the pilot responses. The overall reliability coefficient was 0.8296, indicating good internal consistency. When each item was individually excluded, the coefficient remained between 0.814 and 0.8286, confirming the adequacy of all items.

Ethical approval for this study was obtained from the Institutional Review Board (IRB) of Imam Abdulrahman Bin Faisal University (IRB# 2023-01-233), Dammam, Saudi Arabia.

### 2.3 Statistical analysis

The collected data were analyzed using SPSS v28 (IBM Corp., Armonk, NY, USA).

Descriptive analyses were performed to summarize participant characteristics and response patterns. Categorical variables were expressed as frequencies and percentages. The Chi-square test was used to assess associations between independent categorical variables. A *P*-value less than 0.05 was considered statistically significant.

## 3 Results

A total of 234 pediatric practitioners completed the online questionnaire. Based on years of experience, 101 respondents (43%) were classified as Group A (1–9 years of experience), while 133 (57%) were in Group B (10 or more years of experience). Table 1 summarizes the demographic and academic characteristics of the participants. Among all respondents, 133 (57%) were male and 101 (43%) were female. Participants' ages ranged from 25 to 69 years, with a mean age of 40 years. The mean age in Group A was 33 years, whereas it was 45 years in Group B.

**Table 1:** The demographic and academic characteristics of participants.

Characteristics	Group A *N* = 101 (43%)	Group B *N* = 133 (57%)	Total *N* (%)
Sex			
Male	46 (46)	87 (65)	133 (57)
Female	55 (54)	46 (35)	101 (43)
Age (years)			
Range; Mean	25–52; 33	30–69; 45	
Institution type			
MOH hospitals^a^	60 (59)	65 (48.87)	125 (53)
University hospitals	13 (13)	12 (9.02)	25 (11)
Private hospitals	10 (10)	25 (18.8)	35 (15)
Military hospitals	16 (16)	18 (13.53)	34 (15)
Primary healthcare centers	1 (1)	9 (6.77)	10 (4)
Other	1 (1)	4 (3.01)	5 (2)
Profession			
Pediatric consultants	23 (22.77)	78 (58.65)	101 (43)
Pediatric specialist/registrars	36 (35.64)	44 (33.08)	80 (34)
Senior pediatric residents	25 (24.75)	10 (7.52)	35 (15)
Junior pediatric residents	17 (16.83)	1 (0.75)	18 (8)
Pediatric specialty			
General pediatrics	70 (69.31)	77 (58)	147 (63)
Pediatric emergency	10 (9.9)	15 (11)	25 (11)
Pediatric intensive care	4 (3.96)	11 (8)	15 (6)
Others^b^	17 (16.83)	30 (23)	47 (20)

a MOH, Ministry of Health.

b Other pediatric subspecialty including cardiology, pulmonology, immunology, genetic/metabolic, endocrinology, hematology, nephrology, neonatology, gastroenterology, and rheumatology.

In terms of professional roles, pediatric specialists and registrars constituted the largest subgroup in Group A (36 participants, 36%), followed by senior residents (25 participants, 25%). In contrast, pediatric consultants formed the majority in Group B, accounting for 59% (78 participants). General pediatrics was the most frequently reported area of practice in both groups, cited by 70 participants (69%) in Group A and 77 participants (58%) in Group B. Geographical distribution showed that 66 respondents (28%) were from the Eastern Region, 63 (27%) from the Riyadh Region, and 59 (25%) from the Western Region, with the remainder practicing in other regions across the country. Half of the participants reported working in Ministry of Health (MOH) hospitals, while the other half were distributed across university, private, and military institutions. However, due to the relatively small and uneven numbers of participants across these institutional categories, no meaningful statistical comparison of IIH awareness by institution type could be performed.

Overall, 61 respondents (26%) reported having encountered patients with IIH in their clinical practice, while 173 (74%) had not. The proportion of those with such experience was similar in both groups, with 27 participants (26.73%) in Group A and 34 (25.56%) in Group B, indicating comparable exposure to IIH cases.

The general knowledge level regarding IIH was moderate. The overall correct response rate was 73%, with 57% were among those who had completed the pediatric training, i.e., consultants and specialists, and 16% among pediatric residents currently in training.

Table 2 provides a detailed comparison of IIH-related knowledge across both groups. Nearly all participants (98%, *n* = 229) correctly identified the definition of IIH. Awareness of the equal incidence of IIH between males and females during the prepubertal period was limited to 39% of respondents. This knowledge was more common among pediatricians in group B, where 45% answered correctly, compared to 31% in group A (*p* = 0.025). Regarding risk factors, 207 participants (88%) recognized obesity as a relevant factor. While the comparison between groups did not reach statistical significance (*p* = 0.072), the trend suggested better recognition in group B.

**Table 2:** Comparative answers provided by participants regarding the IIH-related questions.

No.	IIH-related questions	Group A % (*N*)	Group B % (*N*)	*P*-value*
1.	IIH is defined as raised intracranial pressure in the absence of a brain parenchymal lesion, vascular malformation, hydrocephalus, or CNS infections.	99% (100)	97% (129)	0.290
2.	IIH incidence in prepubertal children shows that females and males are affected equally.	31% (31)	45% (60)	0.025**
3.	A risk factor for developing IIH in pubertal children is obesity.	84% (85)	92% (122)	0.072
4.	The diagnostic criteria for IIH are known as The Modified Dandy Criteria.	43% (43)	48% (64)	0.398
5.	The most common symptom reported in IIH is headache.	90% (91)	98% (131)	0.003**
6.	The headache associated with IIH is most often severe in the morning and can be exacerbated by maneuvers such as Valsalva, lying supine, bending over, or coughing.	81% (82)	87% (116)	0.205
7.	Assessment of children with suspicion of IIH should include an ophthalmic examination including pupillary and ocular muscle movements, color vision testing, visual acuity, funduscopic examination, and visual field testing.	94% (95)	93% (124)	0.798
8.	Optic disc edema is a required finding to diagnose IIH in children.	34% (34)	24% (32)	0.105
9.	Visual acuity is often not affected and should not be used as a method to exclude the diagnosis of IIH.	50% (51)	49% (65)	0.805
10.	IIH could be associated with cranial nerve VI palsy.	60% (61)	62% (83)	0.754
11.	In children with suspected IIH, an MRI should be done to exclude other causes.	91% (92)	94% (125)	0.397
12.	One of the most important tools for the diagnosis of IIH is a lumbar puncture with opening pressure measurement.	91% (92)	86% (114)	0.209
13.	The opening pressure when performing a lumbar puncture could be affected by improper positioning or sedation status.	72% (73)	82% (109)	0.077
14.	Management of a patient with IIH often requires a multidisciplinary approach with neurology and ophthalmology.	90% (91)	99% (132)	0.001**
15.	In pubertal children with IIH weight loss is the only modifiable risk factor shown to affect the intracranial pressure and prevent recurrence.	46% (46)	56% (74)	0.125
16.	The first line of pharmacologic management to treat IIH is Acetazolamide, and the second line is Furosemide.	71% (72)	74% (99)	0.590
17.	In IIH cases with severe visual deficits at presentation, corticosteroids are used in conjunction with acetazolamide.	61% (62)	65% (86)	0.606
18.	In children with IIH that are resistant to pharmacologic management or intolerance exists, there are surgical options to treat IIH.	71% (72)	71% (94)	0.918
19.	Untreated IIH could lead to permanent vision loss and chronic pain.	82% (83)	88% (117)	0.213
20.	After resolution of IIH symptoms there is a risk of recurrence.	77% (78)	81% (108)	0.455

IIH, Idiopathic intracranial hypertension; CNS, central nervous system.

* Using Chi-square test.

** Indicates statistically significant difference.

Headache was identified as the most common symptom of IIH by 222 participants (95%). This recognition differed by experience, with 90% of Group A and 98% of Group B identifying it correctly (*p* = 0.003). Knowledge of the typical features of IIH-related headache was also relatively high, with 198 participants (85%) responding correctly, although no significant difference was found between the two groups.

Only 107 participants (46%) were aware of the modified Dandy criteria for diagnosing IIH. Additionally, 66 respondents (28%) incorrectly believed that optic disc edema is part of the diagnostic criteria, and 90 (38%) were unaware of the potential association with sixth cranial nerve palsy. However, the majority acknowledged the importance of diagnostic investigations: 219 participants (94%) recognized the role of ophthalmologic assessment, and 206 (88%) identified both MRI and lumbar puncture with opening pressure measurement as essential diagnostic tools.

Regarding management, 120 participants (51%) correctly identified weight loss as the only modifiable risk factor to reduce recurrence. Knowledge of treatment options was more robust, with 171 respondents (73%) aware of the first- and second-line pharmacologic therapies, and 166 (71%) recognizing the availability of surgical interventions for refractory cases. Most participants acknowledged that managing IIH requires a multidisciplinary approach, particularly involving neurologists and ophthalmologists. This recognition was more common among those with greater experience: 99% of Group B supported this approach, compared to 90% in Group A (*p* = 0.001).

## 4 Discussion

### 4.1 General knowledge and clinical presentation

This study aimed to assess pediatricians’ knowledge and awareness of IIH in children within Saudi Arabia. The majority of participants reported no prior clinical encounter with IIH, which may partly explain the overall moderate level of familiarity with the condition. Notably, knowledge gaps were also observed among pediatricians who had previously managed patients with IIH.

While nearly all respondents accurately identified the general definition of IIH (1), fewer than half were aware that IIH occurs with equal frequency among prepubertal males and females. Pediatricians with longer clinical experience demonstrated a better understanding of this epidemiologic pattern, although only 45% answered correctly. This misconception likely stems from the well-known female predominance seen in adults and post-pubertal children (9). Interestingly, prior observational studies from the region have shown that most pediatric IIH cases occurred in the prepubertal age group (6, 10). Failure to recognize this demographic distribution particularly among young boys may contribute to diagnostic delays. In addition, IIH in younger children may present without headache, and diagnosis is often prompted after detecting optic disc swelling, underscoring the need for greater awareness of such atypical presentations (11).

The majority of participants correctly identified obesity as a risk factor for IIH, especially those with longer experience. Several studies have established a strong association between obesity and IIH in post-pubertal children, similar to adults. However, this relationship is less consistent in prepubertal children (10, 12). In fact, asymptomatic presentations of IIH have been reported more frequently in younger children, particularly in males and those without obesity (13). This further underscores the importance of tailoring clinical suspicion to age-specific risk profiles.

Regarding symptomatology, headache is well-recognized as the most common presenting symptom in pediatric IIH (2), and this was correctly identified by most participants. However, fewer respondents were familiar with the typical features of IIH-related headache. Although headache characteristics in children are often under-reported in the literature, clinical reviews suggest that these headaches are commonly exacerbated by actions that increase ICP, such as Valsalva maneuvers or positions like lying flat or bending forward (14, 15). Sager et al. reported that headache aggravated by leaning forward or restricting play was significantly associated with IIH and elevated CSF pressure in children (16).

Awareness of non-headache manifestations was limited. In particular, many participants failed to recognize sixth cranial nerve palsy, the most commonly reported cranial nerve deficit in IIH (17). These knowledge gaps may impair pediatricians' ability to identify clinical red flags in children presenting with headache, potentially resulting in delayed diagnosis and prolonged patient discomfort.

### 4.2 Knowledge regarding the diagnostic evaluation of IIH

While most participants were aware of the importance of neuroimaging, lumbar puncture, and CSF opening pressure measurement, fewer demonstrated familiarity with the complete modified.

Dandy criteria and its diagnostic components. These criteria remain the gold standard for diagnosing IIH, serving to exclude secondary causes of raised ICP (3). Neuroimaging plays a central role in the diagnostic process by ruling out other etiologies such as hydrocephalus, intracranial mass, or abnormal meningeal enhancement. More recent updates to the diagnostic criteria include specific MRI findings that support a diagnosis of IIH in the absence of papilledema. These include an empty sella, flattening of the posterior globe, distension of the peri-optic subarachnoid space, and transverse venous sinus stenosis (9). The presence of any three of these neuroimaging signs has been shown to yield a sensitivity of 64% and a specificity ranging from 97% to 100% in patients with chronic headache but no papilledema (18). Recognizing these radiologic features is critical for identifying patients with atypical or subtle clinical presentations.

Although participants demonstrated strong knowledge of neuroimaging and lumbar puncture, the limited understanding of diagnostic criteria may hinder accurate confirmation of IIH. This highlights the need for targeted education to reinforce not only the steps in diagnostic workup but also the application and interpretation of the diagnostic criteria.

Participants also acknowledged the importance of ophthalmologic evaluation for suspected IIH cases. However, a considerable proportion, 168 participants (71.79%) incorrectly believed that optic disc edema is essential for diagnosis, despite evidence showing that approximately 17% of patients with IIH may present without papilledema (19). Conversely, false positives are also a concern. In a study by Krishnakumar et al., 4 of 15 children initially suspected to have IIH based on presumed papilledema were ultimately diagnosed with optic disc drusen following detailed ophthalmic assessment, leading to withdrawal of the IIH diagnosis (20). This underscores the necessity of comprehensive ophthalmological evaluation including expert fundoscopy, orbital ultrasonography, and optical coherence tomography to avoid misdiagnosis.

### 4.2 Knowledge regarding the management of IIH

This study revealed notable gaps in pediatricians' awareness of management strategies for IIH. Although the majority of participants correctly identified obesity as a risk factor, nearly half did not recognize weight loss as a cornerstone of non-pharmacologic treatment. Given the established correlation between body mass index and cerebrospinal fluid (CSF) pressure, weight reduction remains a critical component of IIH management (22).

Regarding pharmacologic therapy, 73% of respondents were familiar with first- and secondline treatment options. Additionally, 71% were aware of the availability of surgical interventions such as optic nerve sheath fenestration, CSF diversion techniques, and venous sinus stenting. This suggests moderate familiarity with therapeutic modalities, although continuous education is necessary to ensure a comprehensive understanding of treatment selection, escalation, and individualized care. Variable patient responses to therapy underscore the importance of staying updated on all available management strategies.

Previous studies have shown that many patients respond well to initial medical management, with resolution of symptoms and papilledema in most cases (12, 21). However, recurrence remains a concern, with over one-third of patients experiencing relapse within one year of discontinuing treatment (12). Factors such as the severity of papilledema at presentation have been associated with worse long-term outcomes (21). Female sex and disease recurrence have also been identified as predictors of poor visual prognosis (23). In refractory cases where vision is threatened, surgical intervention may be warranted, aligning with adult management guidelines (24, 25).

A multidisciplinary approach involving both pediatric neurologists and ophthalmologists is essential in the evaluation and management of pediatric IIH. Previous studies have shown that patients followed by a multidisciplinary team have lower rates of hospital readmission compared to those managed by individual specialists (21). In our study, years of clinical experience were significantly associated with support for multidisciplinary care. Pediatricians with longer experience may be more inclined toward collaborative care, likely due to greater awareness of the limitations of single-specialty management and enhanced access to inter-specialty networks. Promoting this approach among all levels of pediatric practice is critical to improving outcomes in children with IIH.

Most participants in the present study correctly recognized potential complications of IIH, including visual loss and recurrence. However, prognosis is highly dependent on timely diagnosis and coordinated, multidisciplinary management to address modifiable risk factors and minimize long-term morbidity.

These findings highlight the urgent need for targeted educational and training initiatives to improve pediatricians' understanding of IIH diagnosis, treatment, and prognosis. Enhancing knowledge in this area can support earlier identification, optimize care pathways, and reduce preventable complications such as irreversible vision loss. This study provides a valuable starting point for future efforts to improve the clinical management of IIH in Saudi Arabia and may serve as a reference for countries with similar healthcare challenges.

Although this study included a diverse sample of pediatricians representing various geographic regions and healthcare institutions across Saudi Arabia, several limitations should be acknowledged. The relatively small sample size and the use of an online questionnaire distributed via email may limit the generalizability of the findings. Additionally, as with all survey-based research, responses may be influenced by recall bias or social desirability bias and may not fully reflect actual clinical practice behaviors.

Future studies may further explore differences in knowledge and clinical decision-making between pediatricians who have managed IIH cases and those without prior exposure, to better understand how clinical experience influences awareness. In addition, expanding the scope of assessment to include awareness of secondary causes such as endocrine or nutritional disorders, identification of headache red-flag symptoms, and evaluation of access to neuroimaging in resourcelimited settings would provide a more comprehensive understanding of the factors influencing early recognition and management of pediatric IIH.

## 5 Conclusion

This study highlights significant gaps in pediatricians' knowledge and awareness regarding the diagnosis and management of IIH. There is a clear need to strengthen training programs and implement targeted educational initiatives to enhance understanding of IIH among pediatric practitioners. We suggest developing structured workshops, continuing medical education modules, and guideline-based educational resources specifically tailored for pediatricians to address these knowledge gaps. Such efforts are critical to ensuring early recognition, appropriate management, and improved outcomes for affected children and adolescents. Future research involving larger, more representative samples of pediatric healthcare providers is warranted to further assess knowledge levels and guide national strategies for clinical education and awareness.

## Data Availability

The raw data supporting the conclusions of this article will be made available by the authors, without undue reservation.
