# Supplementary material for: Awareness of idiopathic intracranial hypertension in children among pediatricians in Saudi Arabia: a cross-sectional study
Source: Front Pediatr. 2025 Nov 7;13:1675107. doi: 10.3389/fped.2025.1675107 (PMC12634519; doi:10.3389/fped.2025.1675107)
Supplement: Supplementary file 1 [file Datasheet1.pdf]

# Pediatric physician's awareness of IIH Survey Content

## General information:

- Gender
- Age
- Current profession
- Sub-specialty (if applicable)
- Years of work experience in pediatrics
- Type of affiliated institute
- Region of affiliated institute
- Is IIH frequently encountered in your practice.

## Research data: (The following questions are answered with I agree, I disagree, or I don't know)

1. Idiopathic intracranial hypertension (IIH) is defined as raised intracranial pressure in the absence of a brain parenchymal lesion, vascular malformation, hydrocephalus, CNS infections, or the use of some medications.
2. IIH incidence in prepubertal pediatrics shows that females and males are affected equally.
3. A risk factor for developing IIH in pubertal pediatrics is obesity.
4. The diagnostic criteria for IIH is known as The Modified Dandy Criteria.
5. The most common symptom reported in IIH is headache.
6. The headache associated with IIH is most often severe in the morning and can be exacerbated by maneuvers such as Valsalva, lying supine, bending over, or coughing.
7. Assessment of pediatrics with suspicion of IIH should include an ophthalmic examination including pupillary and ocular muscle movements, color vision testing, visual acuity, funduscopic examination, and visual field testing.
8. Around 17% of pediatrics with IIH don't have optic disc edema at presentation.
9. Visual acuity is often not affected and should not be used as a method to exclude the diagnosis of IIH.
10. IIH could be associated with cranial nerve VI palsy.
11. In pediatrics with suspected IIH, an MRI should be done to exclude other causes.
12. One of the most important tools for the diagnosis of IIH is a lumbar puncture with opening pressure measurement.
13. The opening pressure when performing a lumbar puncture could be affected by improper positioning or sedation status.
14. Management of a patient with IIH often requires a multidisciplinary approach with neurology and ophthalmology.
15. In pubertal pediatrics with IIH weight loss is the only modifiable risk factor shown to affect the intracranial pressure and prevent recurrence.
16. The first line of pharmacologic management to treat IIH is Acetazolamide and the second line is Furosemide.
17. If kept untreated IIH could lead to permanent vision loss and chronic pain.
18. In IIH cases with severe visual deficits at presentation, corticosteroids are used in conjunction with acetazolamide.
19. In pediatrics with IIH that is resistant to pharmacologic management or intolerance exists, there are surgical options to treat IIH.
20. After resolution of IIH symptoms there is a risk of recurrence.
